# Machine-learning-based predictive classifier for bone marrow failure syndrome using complete blood count data

**DOI:** 10.1016/j.isci.2024.111082

**Published:** 2024-10-01

**Authors:** Jeongmin Seo, Chansub Lee, Youngil Koh, Choong Hyun Sun, Jong-Mi Lee, Hong Yul An, Myungshin Kim

**Affiliations:** 1 Department of Internal Medicine, Seoul National University Hospital, Seoul, Republic of Korea; 2 Department of Internal Medicine, Seoul National University Bundang Hospital, Seongnam-si, Gyeonggi-do, Republic of Korea; 3 NOBO Medicine Inc., Seoul, Republic of Korea; 4 Center for Precision Medicine, Seoul National University Hospital, Seoul, Republic of Korea; 5 Department of Laboratory Medicine, College of Medicine, The Catholic University of Korea, Seoul, Republic of Korea; 6 Catholic Genetic Laboratory Centre, Seoul St. Mary’s Hospital, College of Medicine, The Catholic University of Korea, Seoul, Republic of Korea

**Keywords:** Hematology, Machine learning

## Abstract

Accurate risk assessment of bone marrow failure syndrome (BMFS) is crucial for early diagnosis and intervention. Interpreting complete blood count (CBC) data is challenging without hematological expertise. To support primary physicians, we developed a predictive model using basic demographics and CBC data collected retrospectively from two major hospitals in South Korea. Binary classifiers for aplastic anemia and myelodysplastic syndrome were created and combined to form a BMFS classifier. The model demonstrated high performance in distinguishing BMFS, with consistent results across different CBC feature sets, confirmed by external validation. This algorithm provides a practical guide for primary physicians to identify BMFS based on initial CBC data, aiding in effective triage, timely referrals, and improved patient care.

## Highlights

• Predictive model for bone marrow failure syndrome using CBC and differential data
• Accurately classifies aplastic anemia and myelodysplastic syndrome
• High performance across feature sets, confirmed by external validation
• Practical guidance for primary physicians to identify BMFS early

## Graphical abstract

**Figure:**
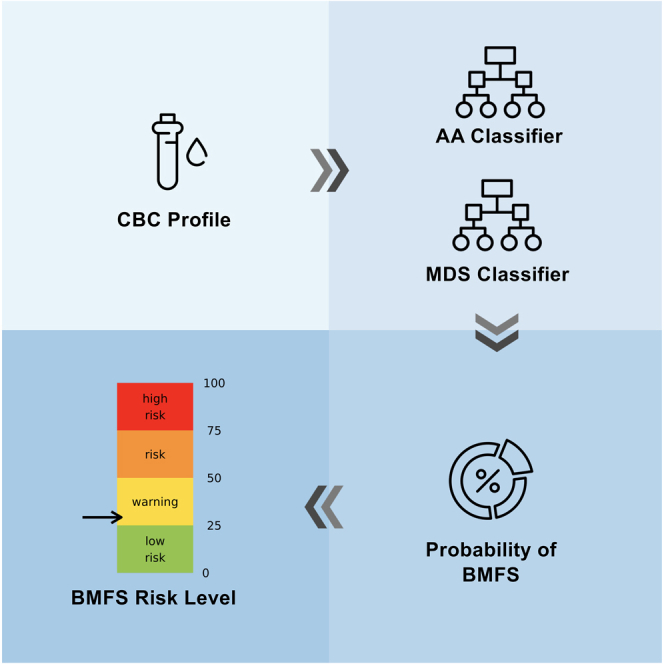

## Introduction

Bone marrow failure syndrome (BMFS) is a heterogeneous collection of hematological diseases characterized by impaired hematopoiesis in one or more lineages and consequent peripheral cytopenia.^1^ BMFS can be broadly classified as either inherited or acquired. Inherited BMFS occurs due to specific germline mutations in hematopoietic stem or progenitor cells, resulting in constitutional syndromes, such as Fanconi anemia, dyskeratosis congenita, and Shwachman-Diamond syndrome. Conversely, acquired BMFS results from extrinsic damage to hematopoietic stem cells caused by drugs, radiation, infection, or immunological diseases. Aplastic anemia (AA), myelodysplastic syndrome (MDS), and paroxysmal nocturnal hemoglobinuria (PNH) are the primary causes of acquired BMFS.^1^^,^^2^^,^^3^

BMFS commonly presents as cytopenia detected through a complete blood count (CBC). CBC, one of the most common laboratory tests ordered by clinicians, provides information regarding peripheral blood cell counts, sizes, and population data collected through automated hematologic analyzers.^4^^,^^5^ To definitively diagnose BMFS, timely referral to hematologic specialists and further explorations such as bone marrow (BM) examination, flow cytometry, cytogenetics, and molecular assays are required.^3^^,^^4^ However, making this referral decision is challenging because of the prevalence of cytopenia, while actual BMFS cases are rare. Certain CBC features (CF), such as the mean corpuscular volume (MCV) and red cell distribution width (RDW), can provide valuable insights for detecting BMFS.^6^^,^^7^ Nevertheless, this interpretation may be difficult for general practitioners without hematological expertise. Consequently, there is a pressing need for complementary tools that can distil intricate hematological data into actionable insights, empowering non-hematological healthcare providers to enhance diagnostic accuracy and referral decisions.

Recently, machine-learning-based approaches have emerged as promising solutions in hematology for disease subtype classification, risk stratification, and treatment decisions.^8^^,^^9^^,^^10^^,^^11^ These algorithms learn from vast datasets and combine diverse clinical and laboratory parameters to recognize intricate patterns that may be missed during human observations.^12^ Several machine-learning-based algorithms have been developed to diagnose BMFS using demographic, laboratory, and genomic data. However, most studies have limited their diagnostic scope to MDS alone or have utilized comprehensive test results such as BM examination, PNH clones, karyotype, and telomere length.^13^^,^^14^^,^^15^^,^^16^ To the best of our knowledge, no study has reported a classifier to diagnose various BMFS diseases using basic demographics and CBC data alone, which is the limited information available on primary clinical encounters with patients.

To address this unmet clinical requirement, we present a machine-learning-based classifier designed to assist primary physicians in the early identification of potential BMFS cases. Our algorithm integrates age, sex, and CBC values to generate an effective risk assessment of the likelihood of BMFS, enabling a more effective triage, timely referrals, and improved patient care.

## Results

### Dataset summary

**Figure 1:**
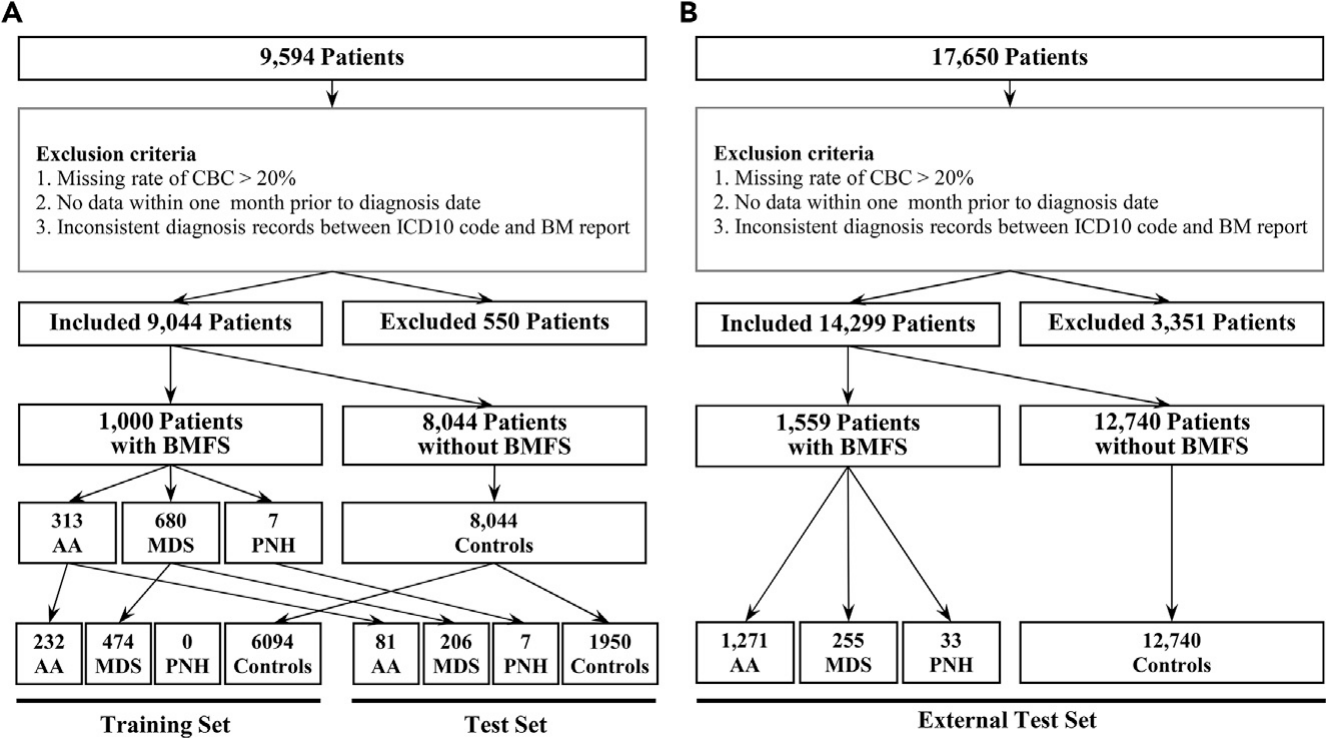
Flowchart of patient selection and assignment to training, test, and external test set (A) In SNUH dataset, 6,800 patients (232 of AA, 474 of MDS, and 6,094 of control) are involved in classifier training, while the remaining patients (81 of AA, 206 of MDS, 7 of PNH, and 1,950 of control) are involved in classifier evaluation. (B) In CMC dataset, 1,559 patients with BMFS (1271 of AA, 225 of MDS, and 33 of PNH) and 12,740 patients without BMFS are involved in the external validation. BMFS: bone marrow failure syndrome, AA: aplastic anemia, MDS: myelodysplastic syndrome, PNH: paroxysmal nocturnal hemoglobinuria.

**Figure 2:**
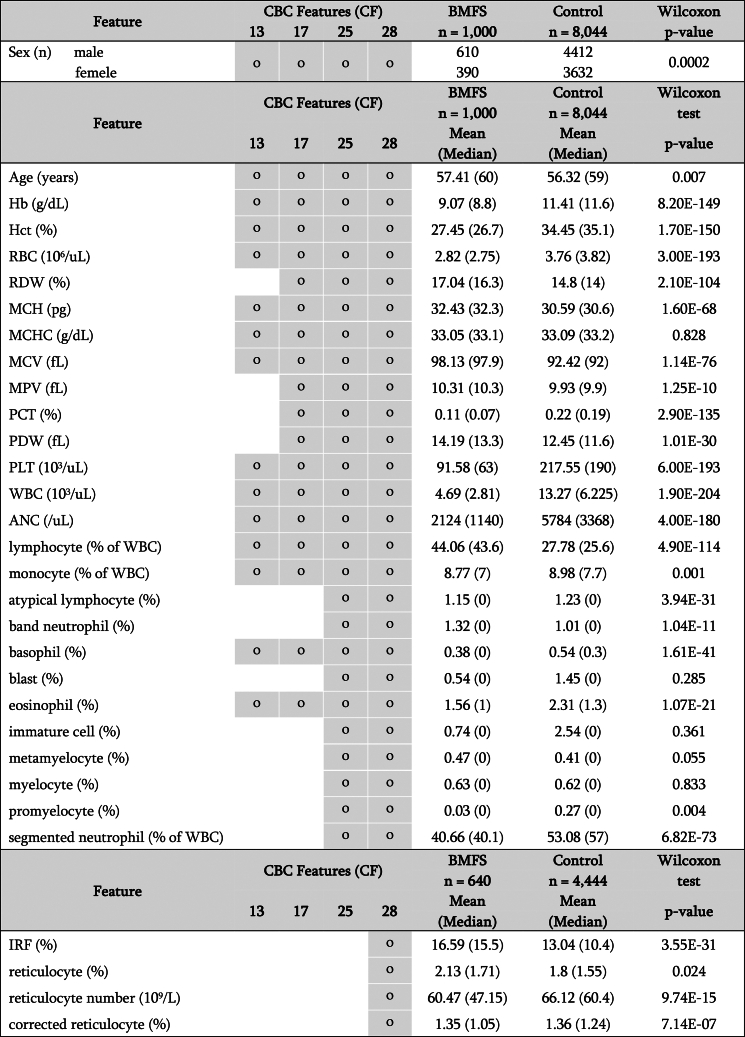
CBC characteristics of the BMFS patients and control group in SNUH dataset The feature set column indicates whether the corresponding feature is included in the model. The numbers in the sex row represent the number of patients, and the remaining numbers represent the mean (and median) values of each feature. Corrected reticulocyte is calculated as (patient’s Hct/45) ∗ reticulocyte count (%), assuming a normal Hct of 45%. *p* values for sex were calculated using Fisher’s exact test between the control group and BMFS group. The other *p* values were calculated using Wilcoxon test between the control group and BMFS group.

The Seoul National University Hospital (SNUH) dataset comprised 9,044 patients, including 1,000 patients with BMFS (313 with AA, 680 with MDS, and 7 with PNH) and 8,044 controls (Figure 1A). Similarly, the Seoul St. Mary’s Hospital of the Catholic Medical Center (CMC) dataset comprised 14,299 patients including 1,559 BMFS cases (1,271 with AA, 255 with MDS, and 33 with PNH), and 12,740 controls (Figure 1B). Among them, a subset of 895 BMFS cases (718 with AA, 155 with MDS, and 22 with PNH) and 2,503 controls had records of reticulocyte features (Figure S1B). Figure 2 and Table S1 present the CF sets used in the classifiers and their summaries.

Notably, three reticulocyte features (immature reticulocyte fraction, reticulocytes, and reticulocyte number) were not routinely tested in either SNUH or CMC. In the SNUH dataset, 640 BMFS cases (195 AA, 440 MDS, and 5 PNH) and 4,444 controls had CBC records for reticulocyte features (Figure S1A). In the CMC dataset, 895 BMFS cases (718 AA, 155 MDS, and 22 PNH) and 2,503 controls also had records of these reticulocyte features (Figure S1B). Therefore, the development of 28 CF-based classifiers was conducted using these subsets of datasets.

### Performance of AA and MDS classifiers

**Figure 3:**
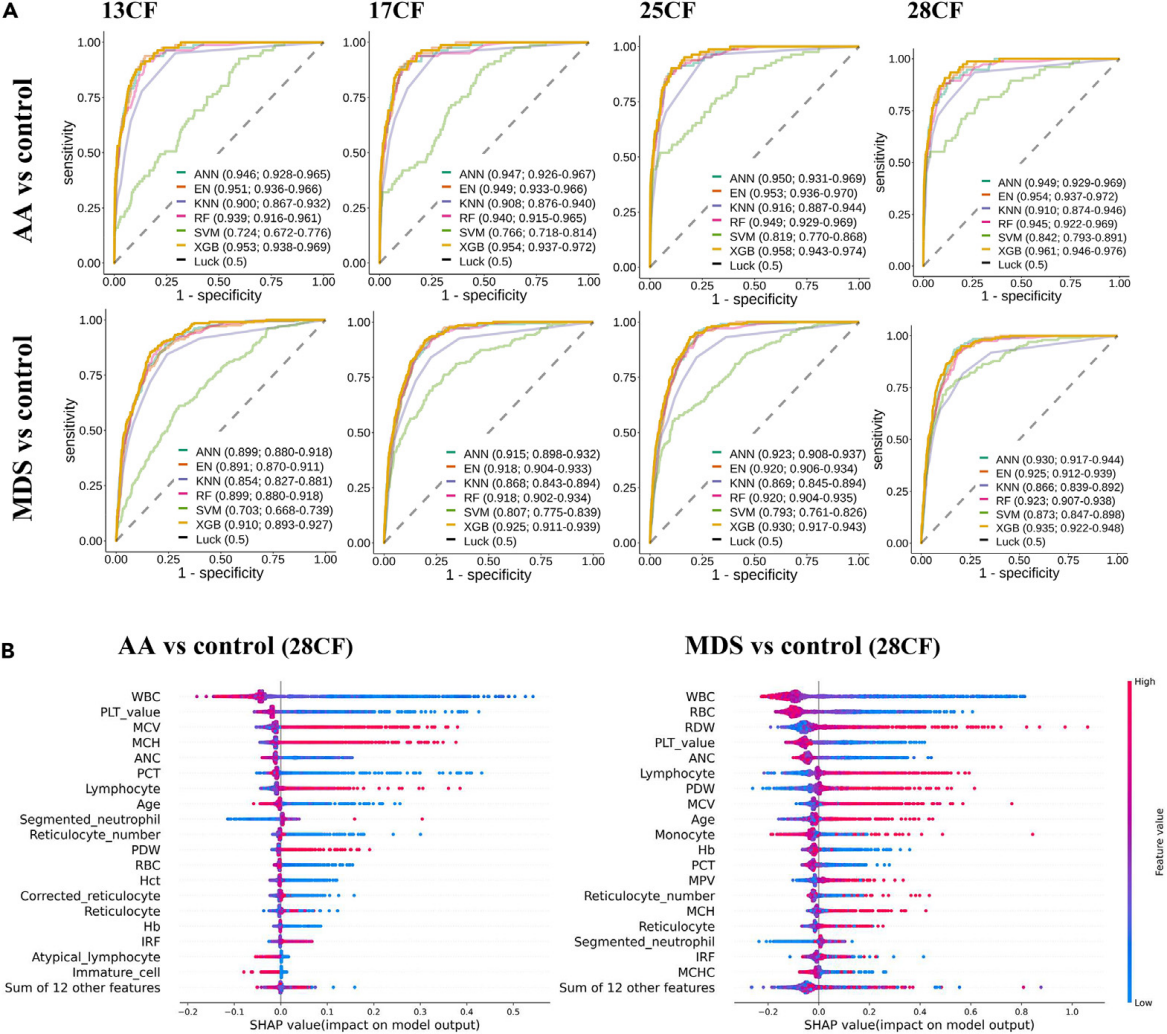
Results of AA and MDS classifiers (A) ROC curves depicting the performance of six machine learning models in classifiers for AA and MDS. The AUROC values and their corresponding 95% confidence intervals are shown in the figure legends. XGBoost consistently exhibited the highest AUROC across all feature sets and in both the AA and MDS classifiers. ANN: artificial neural network, EN: elastic net, KNN: K-nearest neighbor, RF: randome forest, SVM: support vector machine, XGB: XGBoost (B) Feature importance of the AA and MDS classifiers using 28CF. The F scores indicate the frequency of feature occurrence within the decision trees included in XGBoost. The results for the other CF sets are provided in Figure S3.

Missing values were successfully imputed compared to conventional imputation approaches using the mean or median values, as shown in Figure S2. The training set was used to generate the AA and MDS classifiers, which were evaluated using a test set. Among the six machine learning models examined, XGBoost achieved the highest AUROC for the classification of AA and MDS across all CF sets, with AUROCs of 0.953, 0.954, 0.958, and 0.961 for the AA classifier and 0.910, 0.925, 0.930, and 0.935 for the MDS classifier in the 13CF, 17CF, 25CF, and 28CF sets, respectively (Figure 3A). These XGBoost classifiers are used to access the final BMFS probabilities. Feature importance analysis revealed that white blood cells and platelets were the most important factors in predicting AA, while white blood cells and red blood cells were the most important factors in predicting MDS (Figures 3A and S3). In the 28CF-based classifiers, it is worth noting that the number of immature reticulocytes emerged as an important feature, ranking above the middle in feature importance.

### Performance of BMFS classifier

**Figure 4:**
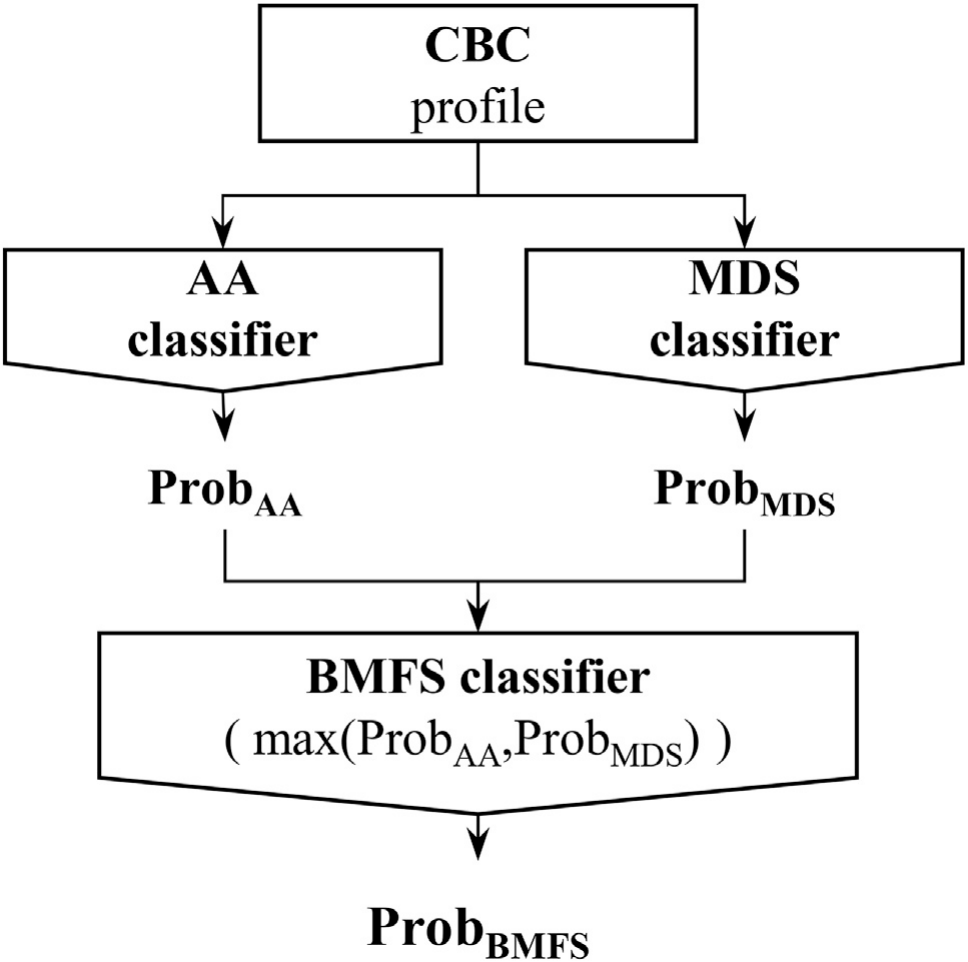
Architecture of BMFS Classifier For each given CBC profile, the AA and MDS classifiers are used to calculate the probabilities of having AA and MDS, respectively. The probability of BMFS is then determined by selecting the maximum output probabilities from the AA and MDS classifiers.

**Figure 5:**
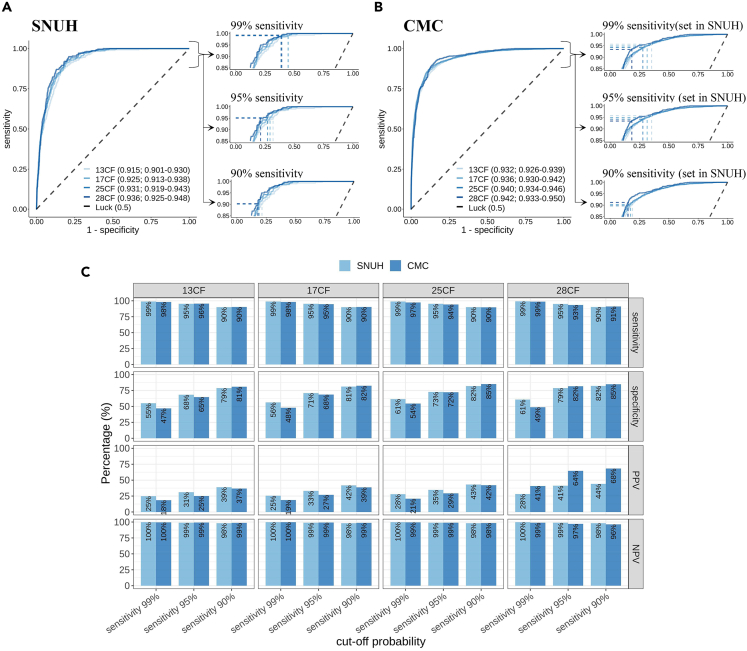
Performance evaluation of BMFS classifiers ROC curves of BMFS classifiers calculated from the (A) test set and (B) external test sets. Each color represents used CF sets in the classifiers. The right panels show the cut-off probabilities that achieve 99%, 95%, and 90% sensitivity, along with the corresponding specificity. (C) Sensitivity, specificity, positive predictive value (PPV), and negative predictive value (NPV) in test and external test sets. Each metric was calculated using the cut-off probabilities set in the SNUH dataset.

The BMFS probabilities of the test set were provided by the maximum output probabilities of the AA and MDS classifiers (Figure 4). Our BMFS classifier performed well, achieving AUROCs of 0.915, 0.925, 0.931, and 0.936 for the multiple CF sets (Figures 5A, S4, S5, and Table S2). As expected, the classifier’s performance improved when more CBC features were included. The class imbalance was addressed by assigning a weight to the case group during model training (Figure S6).

Especially in the 25CF set, the cut-off probability for offering 99.0% sensitivity resulted in 61.4% specificity, 27.9% PPV, and 99.8% NPV, whereas the cut-off probability for achieving 95.2% sensitivity resulted in 72.8% specificity, 34.6% PPV, and 99.0% NPV (Figure 5C). The cut-off probability determined using the Youden index yielded a sensitivity of 92.9%, specificity of 80.0%, PPV of 41.2%, and NPV of 98.7% (Table S3A). Other confusion matrix results are shown in Table S3A. All seven patients with PNH were accurately predicted to have BMFS in sensitivity settings equal to or greater than 95%, indicating the high sensitivity of the classifier in detecting PNH cases (Table S4A).

In the external validation using the CMC dataset, the AA and MDS classifiers showed remarkable performance, with AUROC values of 0.947 and 0.943, respectively, for the 25CF-based classifier (Figure S7). Similarly, the BMFS classifier achieved AUROCs of 0.932, 0.936, 0.940, and 0.942 for 13CF, 17CF, 25CF, and 28CF datasets, respectively (Figure 5B). Especially in 25CF set, the classifier showed a sensitivity of 89.7%–97.4% and specificity of 54.5%–84.9% when employing the cut-offs that offer sensitivity ranging from 90% to 99% in SNUH dataset (Figure 5C, and Table S3B). Additionally, the prediction result of patients with PNH also showed 81.8%–97.0% sensitivity at the same cutoffs (Table S4B).

### Comparison with architectures of conventional classifiers

The limited discriminatory power between AA and MDS posed a challenge in determining whether to consider them separate or combined classes during model development (Figure S8). Our classifier architecture, consisting of classifiers for each disease, exhibited a significantly higher AUROC than both the binary ([AA + MDS] vs. control) and multiclass (AA vs. MDS vs. control) approaches (AUROC when using 25 CF sets = 0.914 and 0.922; *p* < 0.001 and <0.01, respectively) (Figures S9A and S10). These findings are consistent with the external validation set, in which our architecture consistently outperformed both approaches (AUROC when using the 25CF set = 0.929 and 0.934; *p* < 0.001 for both) (Figure S9B). This demonstrates the ability of the proposed model to effectively address the ambiguity associated with class assignments during model development.

### Web-based application

We developed a user-friendly web-based application that enables clinicians and researchers to assess BMFS risk in patients using their CBC data (see Data Sharing Statement). To generate the risk estimate, users provided age, sex, and CBC data consisting of 13CF, 17CF, 25CF, or 28CF. By inputting this information, users receive the BMFS probability score (ranging from 0 to 100%), risk score (ranging from 0 to 100), and risk-level classification (low risk, warning, risk, or high risk) to support clinical decision-making. Detailed documentation of how to use the web-based application is available on the portal.

## Discussion

We successfully developed predictive model for BMFS using age, sex, and CBC data. The XGBoost classifiers achieved high accuracy in distinguishing AA and MDS from the control groups. The combined BMFS classifier exhibited a robust performance, and it was thoroughly validated using an independent dataset. Additionally, we developed a user-friendly web-based application to enable easy access. These findings highlight the potential of our model as a reliable BMFS risk assessment tool for primary physicians encountering patients with cytopenia to aid early triage and timely referral to improve patient care.

Historical attempts to diagnose MDS using CBC profiles date back to 1987 with the emergence of automated analyzers.^17^^,^^18^ A notable example is the scoring system suggested by Buckstein et al.^16^ to assess the risk of MDS. By incorporating age, MCV, lactate dehydrogenase, and RDW, the post-test likelihood of an MDS diagnosis increases from 12% to 48%.^6^^,^^7^ Subsequently, advanced analyzers have been introduced using advanced detection methods and additional CBC parameters to provide more nuanced diagnostic clues.^19^^,^^20^^,^^21^ For instance, Boutault et al.^22^ formulated the MDS-CBC score by incorporating MCV, absolute neutrophil count, and feature called Ne-WX, which represents structural neutrophil dispersion. Elevated Ne-WX levels correlate with the presence of hypo/degranulated neutrophils, reflecting dysplastic cells in MDS. Zhu et al.^23^ proposed that an extended MDS-CBC score with the addition of immature platelet fraction could improve MDS diagnosis. Nevertheless, due to the lack of access to these exploratory features, there is an unresolved need for an adjunctive tool that can be adapted to daily clinical practice.

Several machine-learning-based algorithms for diagnosing MDS have been published. Raess et al.^13^ used CBC and demographic data to develop a classifier with an AUROC of 0.942. Platelet distribution width and RDW were the most discriminating variables. Radhachandran et al.^14^ used demographic data, vital signs, blood chemistry results, and blood cell counts without cell population data. This classifier had an AUROC value of 0.87, and the most important features were age, hematocrit, red blood cell count, and platelet count. Finally, Pozdnyakova et al.^15^ created an algorithm with 26 CBC parameters including features designed for research-use. Using CBC parameters alone, their model had an AUROC of 0.86. With the addition of molecular and demographic data, the AUROC improved to 0.93. In this context, our study is notable because of its wide diagnostic scope and enhanced performance. While utilizing routine CBC features alone, our algorithm successfully predicted not only MDS but also AA and PNH, with an AUROC of 0.915–0.936.

Diagnostic tools for BMFS etiologies other than MDS were scarce until Gutierrez-Rodrigues et al.^16^ developed a machine-learning-based algorithm to differentiate between acquired and inherited BMFS. The difference from our study is that their algorithm targets the initial encounter at hematology-specialized clinics and attempts to predict the etiology of BMFS before genomic assays are reported. Thus, it utilizes more comprehensive parameters, including PNH clones, karyotype, telomere length, BM examination results, and clinical data. In contrast, our algorithm aims to be used in the first clinical encounters in primary care clinics where only CBC data are available.

The strengths of our study arise from several aspects that set it apart from earlier research. First, we included a large number of patients compared to previous studies, which yielded better performance. The algorithm was rigorously validated in a completely independent cohort from a separate hospital. Both SNUH and CMC are major tertiary hospitals that receive numerous referrals from primary care clinics, ensuring that our cohort effectively represents the clinical scenario. Third, various combinations of CBC feature sets were evaluated to reflect diverse clinical settings. Primary care clinics set up CBC tests with varying number of parameters, sometimes excluding certain cell population data or cell size features. To ensure general applicability, we evaluated various feature sets and showed that our algorithm yielded good performance for all. Finally, we developed a user-friendly web-based application that enables easy access to this classifier. Users can provide any number of available CBC parameters because the application automatically selects the closest feature set to calculate the BMFS risk score, enabling its usage in diverse settings.

### Limitation of the study

A limitation of this study lies in the algorithm’s development employing data sourced from a single center, encompassing patients of a single ethnicity, with CBC data generated using restricted types of hematologic analyzers. Additionally, our model did not specifically identify PNH patients due to insufficient patients, which could potentially limit the implementation of our findings in the clinical setting. Thus, we plan to conduct further validation across diverse institutions in various nations to ensure the algorithm’s applicability across different clinical settings.

To the best of our knowledge, this is the first machine-learning-based predictive classifier of BMFS using basic demographics and CBC features alone, which is the limited information provided on primary clinical encounters with patients presenting with cytopenia. Along with the easy-access web-based application, this practical guide will facilitate more effective triage and timely referrals to improve patient care.

## Resource availability

### Lead contact

Further information and requests for resources and materials should be directed to and will be fulfilled by the lead contact, Hong Yul An (anhongyul@gmail.com).

### Materials availability

This study did not generate new unique materials.

### Data and code availability

• **Data:** Data reported in this paper will be shared by the lead contact upon request.
• **Code:** The code generated in this study has been deposited at Zenodo and is publicly available as of the date of publication: https://doi.org/10.5281/zenodo.13756434.
• **Additional information:** We do not possess sequencing data for DNA, RNA, or protein, nor any other genetic information or image data. The classifier included in this paper can be accessed at http://bmfs.genomeopinion.com/'. Any additional information required to reanalyze the data reported in this paper is available from the lead contact upon request.

## Acknowledgments

This work was supported by Institute of Information & communications Technology Planning & Evaluation (IITP) grant funded by the Korea government (MSIT) (2022-0-00333, Multi-faceted analysis of pediatric rare disease Al integrated SW solution development). We would like to thank Editage (www.editage.co.kr) for English language editing.

## Author contributions

J.S.: conceptualization, data curation, formal analysis, investigation, methodology, writing—original draft, writing-review and editing, C.L.: conceptualization, data curation, formal analysis, investigation, methodology, writing—original draft, writing-review and editing, Y.K.: conceptualization, formal analysis, funding acquisition, project administration, supervision, validation, writing-review and editing, C.S.: formal analysis, methodology, supervision, validation, writing-review and editing, J.L.: data curation, formal analysis, investigation, methodology, validation, writing-review and editing, H.Y.A.: conceptualization, formal analysis, investigation, supervision, validation, writing-review and editing, M.K.: conceptualization, formal analysis, methodology, project administration, supervision, validation, writing-review and editing.

## Declaration of interests

C.L., Y.K., C.S., and H.A. are employed at NOBO Medicine Inc.

Y.K., C.S., and H.A. hold stock shares in NOBO Medicine Inc.

## STAR★Methods

### Key resources table

**Table:** 

REAGENT or RESOURCE	SOURCE	IDENTIFIER
**Software and algorithms**		

Python 3.4.9	Python	https://www.python.org
XGBoost 1.5.0	XGBoost	https://xgboost.readthedocs.io/en/stable/python/index.html
scikit-learn 0.20.4	scikit-learn	https://scikit-learn.org
R 3.6.3	R	https://www.r-project.org
missForest 1.4	missForest	https://cran.r-project.org/web/packages/missForest
bestNormalize 1.8.3	bestNormalize	https://cran.r-project.org/web/packages/bestNormalize
pROC 1.18.0	pROC	https://cran.r-project.org/web/packages/pROC/
Code	Zenodo	https://doi.org/10.5281/zenodo.13756434

### Experimental model and study participant details

We retrospectively collected CBC data from patients who underwent BM examinations at Seoul National University Hospital (SNUH) in South Korea from January 2010 to May 2021. To be eligible, patients had to have complete blood count (CBC) results available within one month before or after their BM examination. Consequently, 9,594 patients with CBC profiles were included in the study. All data collection procedures were approved by the Institutional Review Board (IRB) of the SNUH (IRB number 2107-050-1233). To conduct external validation, we collected independent data from the Seoul St. Mary’s Hospital of the Catholic Medical Center (CMC), which consisted of 17,650 patients with CBC profiles who underwent BM examinations between January 2010 and 2021. Figure 2 and Table S1 provide detailed characteristics of the patients’ sex, age, and CBC. No new participants were recruited for this study. All data were obtained from previously collected samples, and no additional samples were gathered specifically for this research. All data were collected in compliance with CMC IRB guidelines and regulations (IRB number KC23WIDI0168).

We extracted 28 features from CBC profiles: hemoglobin, hematocrit (Hct), red blood cell, RDW, mean corpuscular hemoglobin, mean corpuscular hemoglobin concentration, MCV, mean platelet volume, plateletcrit, platelet distribution width, platelet, white blood cell, absolute neutrophil count, lymphocyte, monocyte, basophil, eosinophil, atypical lymphocyte, band neutrophil, blast, immature cell, immature monocyte, large unstained cell, metamyelocyte, myelocyte, normoblast, promyelocyte, segmented neutrophil, immature reticulocyte fraction, reticulocyte, and reticulocyte number. During model development, we used age, sex, and a subset of CBC features. The CBC analyzers are listed in Table S5.

### Method details

#### Pre-processing

We employed ordered quantile normalization to normalize the CBCs of the SNUH dataset, which can be effectively applied to independent datasets.^24^ This method handles values outside the distribution of the model training set through approximation for extrapolation.

This was conducted using the orderNorm function of the bestNormalize R package (ver.1.8.3). The CBCs of the CMC dataset were transformed into the normalized CBCs of the SNUH dataset using the predict function.

To handle missing values, we utilized the MissForest R package (ver.1.4) for inputting missing numeric values (Figure S11).^25^ Notably, three reticulocyte features (immature reticulocyte fraction, reticulocytes, and reticulocyte number) were not routinely tested in either SNUH or CMC. Therefore, we excluded these features from the imputation process and conducted separate analyses using CBC features with no missing values. Additionally, we incorporated a corrected reticulocyte value, which was calculated using the following formula: corrected reticulocyte count (%) = (patient Hct/45) × reticulocyte count (%). This calculation is based on a normal Hct value of 45%.

#### Patient selection

A depiction of the patient selection process is shown in Figure 1. The inclusion criteria were as follows: 1) patients with CBC profiles taken within one month before diagnosis, 2) patients with CBC profiles with a missing rate of less than 20%, and 3) patients whose diagnoses agreed with the results of the BM examination. Consequently, 9,044 patients from the SNUH dataset and 14,299 from the CMC dataset were included in our study (Figure S12).

The case group consisted of patients diagnosed with AA, MDS, or PNH, whereas the control group comprised all other patients. Diagnosis was established according to the International Classification of Diseases (ICD-10) codes (Table S6). In the SNUH dataset, 1,000 patients were assigned to the case group, and 8,044 patients were assigned to the control group (Figure 1A). The case group contained 313, 680, and 7 patients diagnosed with AA, MDS, and PNH, respectively. In the CMC dataset, 1,559 and 12,740 patients were assigned to the case and control groups, respectively. The case group comprised of 1271, 255, and 33 patients diagnosed with AA, MDS, and PNH, respectively (Figure 1B).

#### Model development

We selected one CBC profile per patient to mitigate the potential bias arising from multiple CBC profiles of the same patient. For each patient in the case group, we chose the CBC profile obtained within one month before AA, MDS, or PNH diagnosis (Figure S13). In cases where a patient was diagnosed multiple times with any of these diseases, we selected the first CBC profile to minimize the impact of treatment on patient’s CBC profile. Similarly, the first CBC profile was selected for each patient in the control group.

We devised a BMFS classifier architecture that utilised two distinct binary classifiers for AA and MDS. Specifically, the AA and MDS classifiers differentiated between the AA and control groups and the MDS and control groups, respectively. Finally, the probability of BMFS was calculated as the maximum output probability from the AA and MDS classifiers (Figure 4).

To construct the AA and MDS classifiers, we tested six different machine learning models: artificial neural network, elastic net, k-nearest neighbor, random forest, support vector machine, and XGBoost (XGB). XGB was implemented using the XGBoost package (ver. 1.5.0), while the other models were implemented using the scikit-learn package (ver. 0.20.4) in Python 3.4.9. The best-performing models were selected for the AA and MDS classifiers. For each model, hyperparameter tuning was performed on the training set using 10-fold cross-validation. During this step, the *scale_pos_weight* parameter in XGB and *class_weight* parameter in the other models was involved to adjust the class imbalance.

To ensure the generalizability of our approach across different CBC testing settings, we developed classifiers using feature sets consisting of 13, 17, 25, or 28 CBC features (CF), in addition to sex and age, as outlined in Figure 2 and Table S1. We designated these feature sets as the 13CF, 17CF, 25CF, and 28CF sets. In model development using these sets, we assigned CBCs obtained before 2019 from the SNUH dataset, which accounted for 75% of the total (*n* = 6,800), to the training set. The remaining 25% (*n* = 2,244) were designated as the test set (Figures 1A and S14). Because of the small number of patients with PNH (*n* = 7) (Figure S15), we included them in the test set instead of training a separate PNH classifier. The training set comprised of 232 AA, 474 MDS, and 6,094 control patients, whereas the test set included 81 AA, 206 MDS, 7 PNH, and 1,950 control patients. During the model training phase, we developed AA and MDS classifiers using AA and control and MDS and control pairs from the training set. In the model testing phase, we assessed the BMFS probabilities of the test set using the two classifiers. For an external evaluation, the classifiers were tested using the CMC dataset (Figure 1B). The number of patients in each group involved in the model development using 28CF, which included reticulocyte features, is shown in Figure S1.

In addition, we compared our proposed BMFS classifier architecture with two conventional machine learning architectures: a multiclass classifier that distinguished between the AA, MDS, and control groups (AA vs. MDS vs. control) and a binary classifier that distinguished between the [AA + MDS] and control groups ([AA + MDS] vs. control).

### Quantification and statistical analysis

The classifiers’ performance was evaluated by calculating the AUROC curve using the pROC R package (v1.18.0). Confusion matrices were generated using multiple cut-offs offering 90%, 95%, 99%, or 100% sensitivity, and cutoffs representing the Youden index, where the sum of sensitivity and specificity was maximal.^26^ We calculated sensitivity, specificity, positive predictive value (PPV), and negative predictive value (NPV) for each cut-off. The feature importance of the AA and MDS XGBoost classifiers was calculated using SHAP python package with shap.explainers.Tree function.^27^ Univariate analysis of the CBC features was conducted using the Wilcoxon rank-sum test. We used a two-sided paired DeLong’s test to assess the statistical significance of the performance differences between our classifier architecture and other conventional architectures.
