# Supplementary material for: Machine-learning-based predictive classifier for bone marrow failure syndrome using complete blood count data
Source: iScience. 2024 Oct 1;27(11):111082. doi: 10.1016/j.isci.2024.111082 (PMC11535363; doi:10.1016/j.isci.2024.111082)
Supplement: Document S1. Figures S1–S15 and Tables S1–S6 [file mmc1.pdf]

iScience, Volume *27*

Supplemental information

Machine-learning-based predictive classifier

for bone marrow failure syndrome

using complete blood count data

Jeongmin Seo, Chansub Lee, Youngil Koh, Choong Hyun Sun, Jong-Mi Lee, Hong Yul An, and Myungshin Kim

Supplementary Figures **3**

Supplementary Figure 1. Flowchart of Patient Selection and Assignment to Training, Test, and External Test Set in 28CF-Based Classifier Development. 3

Supplementary Figure 2. Imputation Error 4

Supplementary Figure 3. Feature importance of AA and MDS classifiers 5

Supplementary Figure 4. Calibration Curve 6

Supplementary Figure 5. Net-Benefit Plot 7

Supplementary Figure 6. Impact of Balancing Class Imbalance in Hyperparameter Tuning 8

Supplementary Figure 7. Performance of AA and MDS Classifiers in External Validation 9

Supplementary Figure 8. Performance of Binary Classifier Distinguishing between AA and MDS 10

Supplementary Figure 9. Comparison between Various Architectures of Classifiers 11

Supplementary Figure 10. Performance of Classifier Distinguishing BMFS from Other Disease Subtypes 12

Supplementary Figure 11. Missing Rate of CBC features 13

Supplementary Figure 12. Pie Chart of The Number of Patients 14

Supplementary Figure 13. Selection of Cutoff Period for CBC Data 15

Supplementary Figure 14. Distribution of Diagnosis Dates of SNUH Patients 16

Supplementary Figure 15. Evaluation of an Approach Distinguishing PNH from AA and MDS 17

Supplementary Tables **18**

Supplementary Table 1. CBC Characteristics of the BMFS Patients and Control Group in CMC dataset 18

Supplementary Table 2. Performance Evaluation of Sex-Stratified Models 19

Supplementary Table 3. Confusion matrix and Evaluation metrics 21

Supplementary Table 4. Prediction Results of PNH patients 22

Supplementary Table 5. List of CBC Analysers 23
Supplementary Table 6. ICD-10 Code of Case Group 24

Supplementary Figures

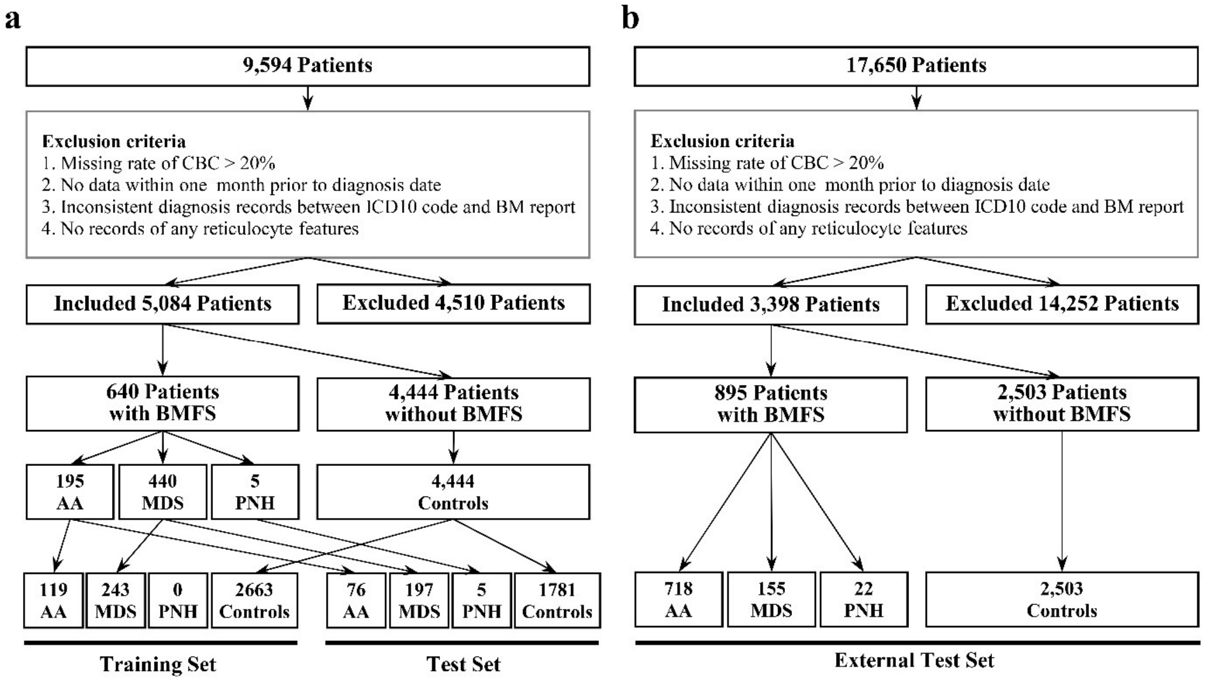

Supplementary Figure 1. Flowchart of Patient Selection and Assignment to Training, Test, and External Test Set in 28CF-Based Classifier Development.

Related to Figure 1. The 28CF-based model only includes patients who have CBC records for all reticulocyte features. (A) In SNUH dataset, 3025 patients (119 of AA, 243 of MDS, and 2663 of control) are involved in classifier training, while the remaining patients (76 of AA, 197 of MDS, 5 of PNH, and 1781 of control) are involved in classifier evaluation. (B) In CMC dataset, 895 patients with BMFS (718 of AA, 155 of MDS, and 22 of PNH) and 2503 patients without BMFS are involved in the external validation of the classifiers. BMFS: bone marrow failure syndrome, AA: aplastic anaemia, MDS: myelodysplastic syndrome, PNH: paroxysmal nocturnal haemoglobinuria.

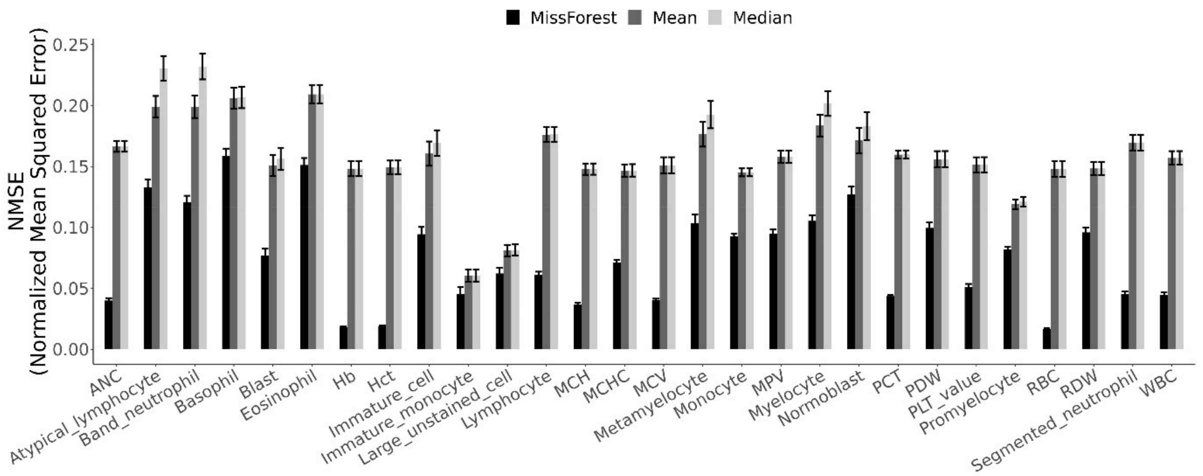

Supplementary Figure 2. Imputation Error

Related to Figure 1. Comparison of imputation methods using the MissForest algorithm, mean, and median. Normalised Mean Squared Errors (NMSE) were calculated using the following steps: (1) Ten simulated datasets were created with 10% missing values in the intact CBC values. (2) Imputation was performed using the MissForest algorithm, mean, or median. (3) For each feature, average NMSE was calculated by comparing the imputed values with the original values. The error bars represent the 95% confidence intervals.

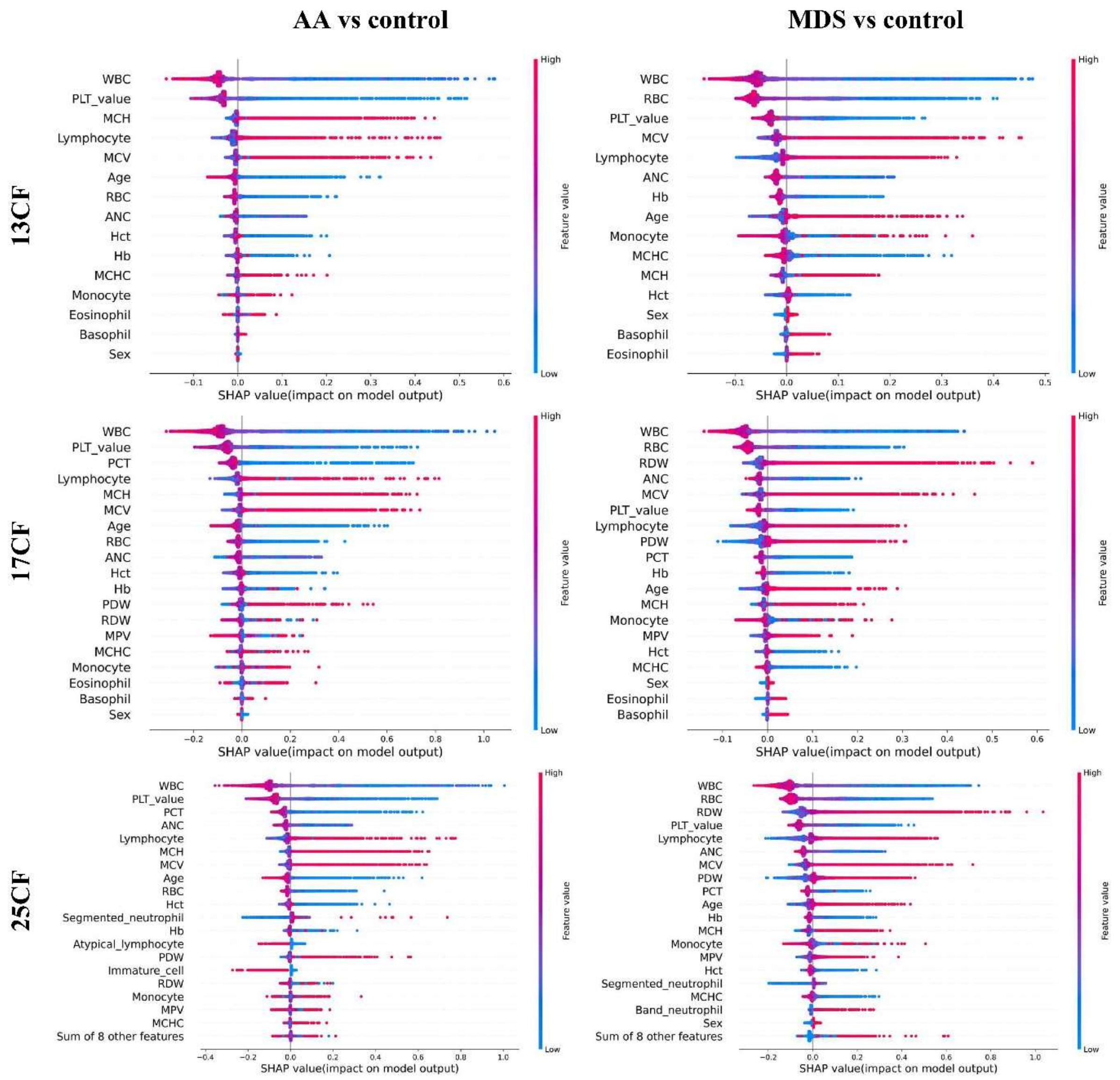

Supplementary Figure 3. Feature importance of AA and MDS classifiers

Related to Figure 2. The F scores indicate the frequency of feature occurrence within the decision trees included in XGBoost. The results of the 28CF-based classifier are provided in Figure 3.

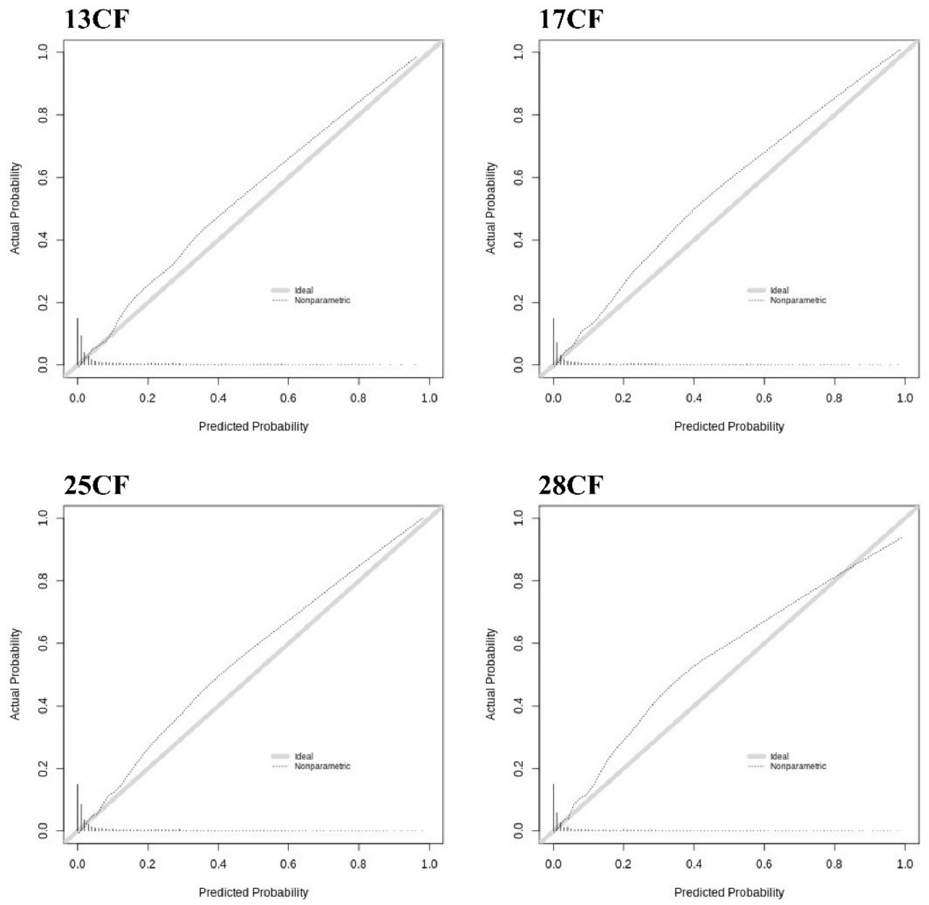

Supplementary Figure 4. Calibration Curve

Related to Figure 3. Calibration curves representing the relationship between predicted probabilities and observed outcomes for BMFS classifiers across different CF sets. These curves were generated using the val.prob function from the rms library in R, which provides a graphical representation of the calibration performance of the models. The x-axis represents the predicted probabilities, while the y-axis represents the observed proportions of positive outcomes.

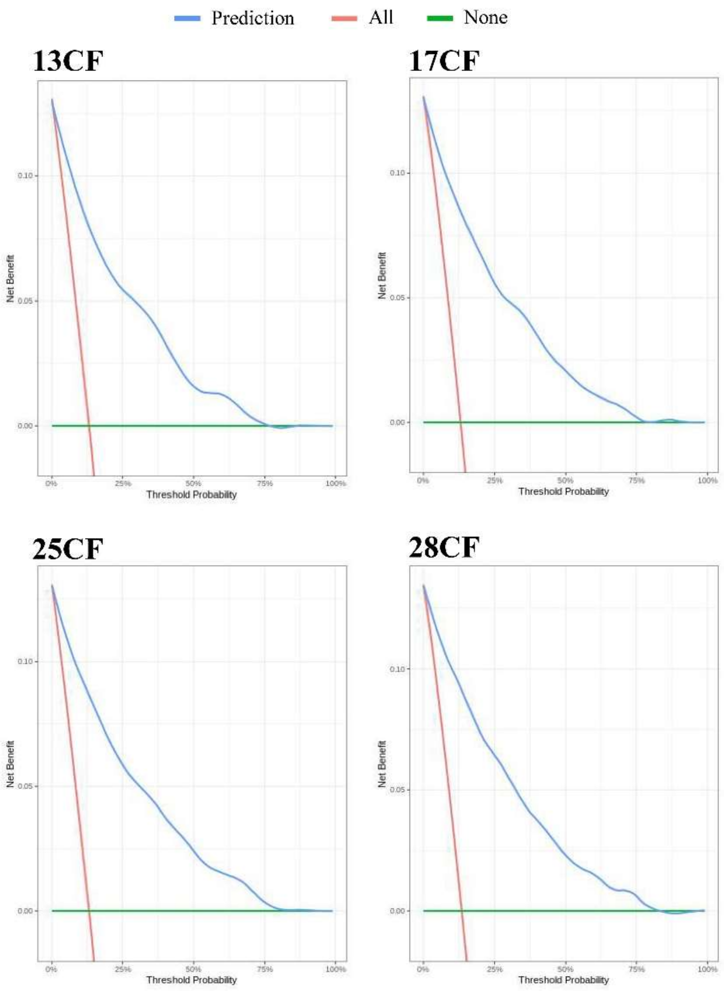

Supplementary Figure 5. Net-Benefit Plot

Related to Figure 3. Net-Benefit plot representing the net benefit of predicting BMFS patients across different CF sets. These plots were generated using the dca function from dcurves rms library in R, which provides a visual assessment of the clinical utility of the models. The x-axis represents the threshold probability, while the y-axis represents the net benefit, accounting for the trade-off between true positives and false positives.

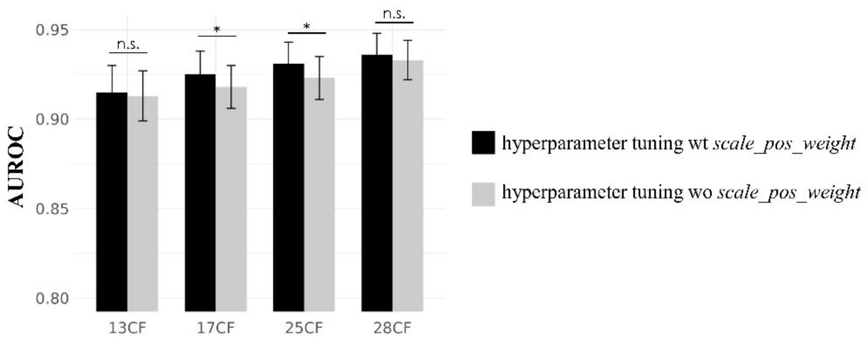

Supplementary Figure 6. Impact of Balancing Class Imbalance in Hyperparameter Tuning

Related to Figure 3. Comparison of AUROC between models with and without the scale_pos_weight parameter during hyperparameter tuning. P-values were calculated using the paired DeLong's test (* < 0.05).

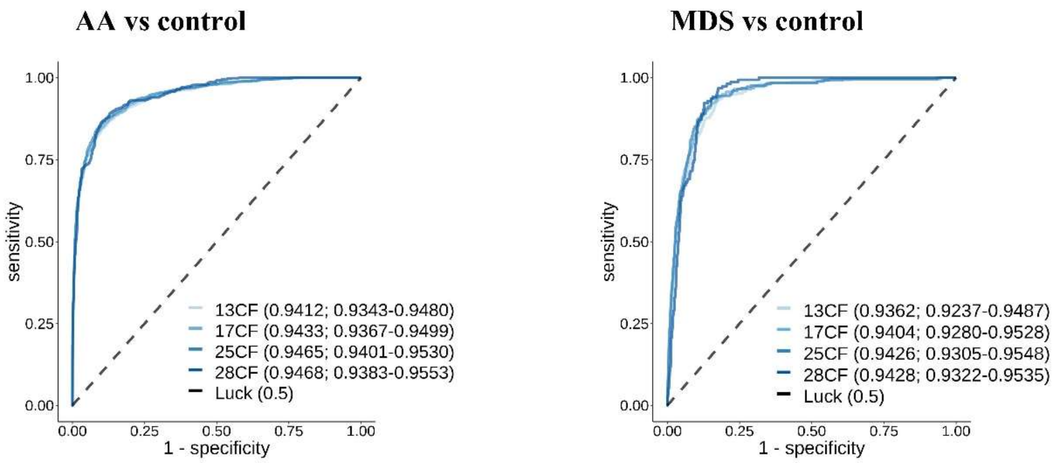

Supplementary Figure 7. Performance of AA and MDS Classifiers in External Validation

Related to Figure 2. ROC curves calculated using CMC dataset, illustrating the performance of the classifiers for AA and MDS using each CF set. The AUROC values and their corresponding 95% confidence intervals are shown in the figure legends.

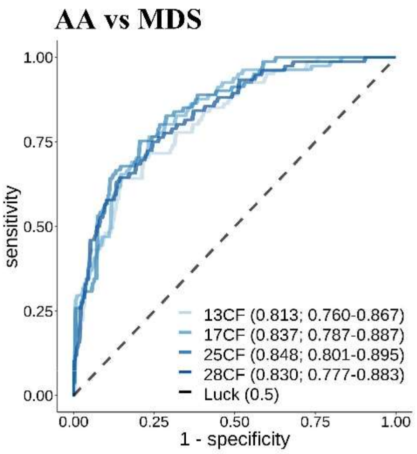

Supplementary Figure 8. Performance of Binary Classifier Distinguishing between AA and MDS

Related to Figure 2. ROC curves depicting the performance of binary classifier distinguishing between AA and MDS in the SNUH dataset. The area under the ROC curve (AUROC) ranges from 0.813 to 0.848, indicating a limited discriminatory power between AA and MDS using CBC data.

a SNUH

b CMC

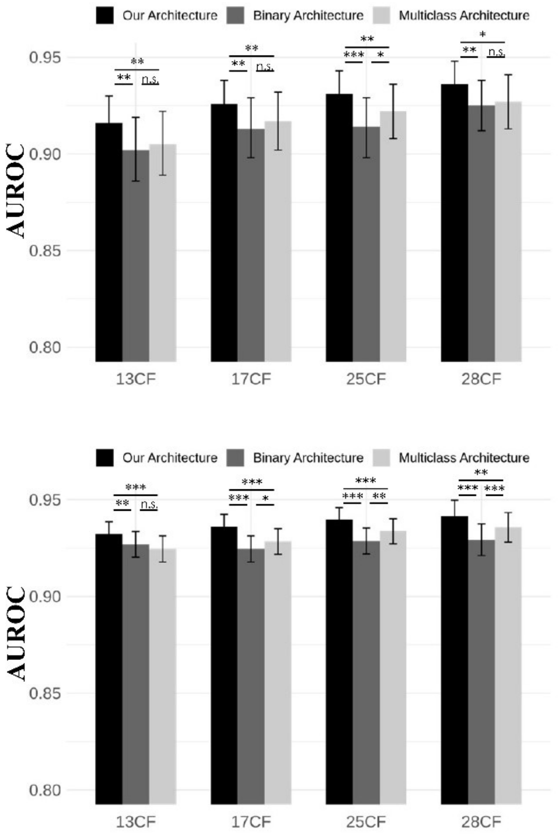

Supplementary Figure 9. Comparison between Various Architectures of Classifiers

Related to Figure 3. The AUROCs of our classifier, the binary ([AA+MDS] vs control) classifier, and the multiclass (AA vs MDS vs control) classifier are compared in (A) SNUH and (B) CMC datasets. Our approach demonstrates superior performance compared to the conventional architectures. It is noted that PNH was excluded from these analyses due to a small number of patients available for training the multiclass classifier with four classes (AA vs MDS vs PNH vs control). P values are calculated using the paired DeLong's test. * < 0.05, ** < 0.01, *** < 0.001.

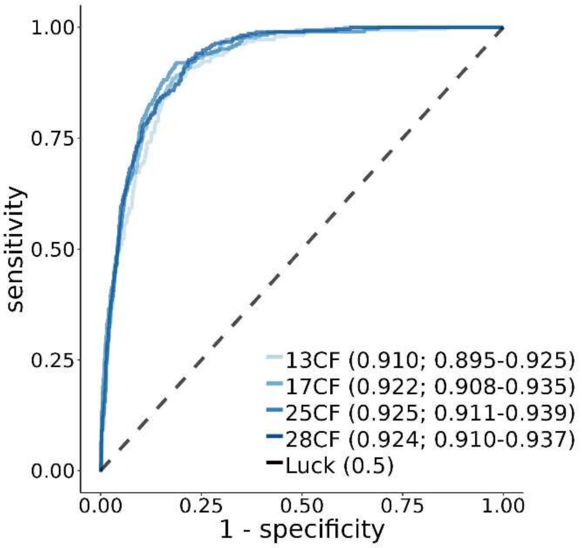

Supplementary Figure 10. Performance of Classifier Distinguishing BMFS from Other Disease Subtypes

Related to Figure 3. ROC curves depicting the performance of multiclass classifier (AA vs MDS vs lymphoma vs leukaemia vs multiple myeloma vs the others) (Supplementary Table). BMFS probabilities are calculated by summing AA and MDS probabilities. The area under the ROC curve (AUROC) ranges from 0.910 to 0.924.

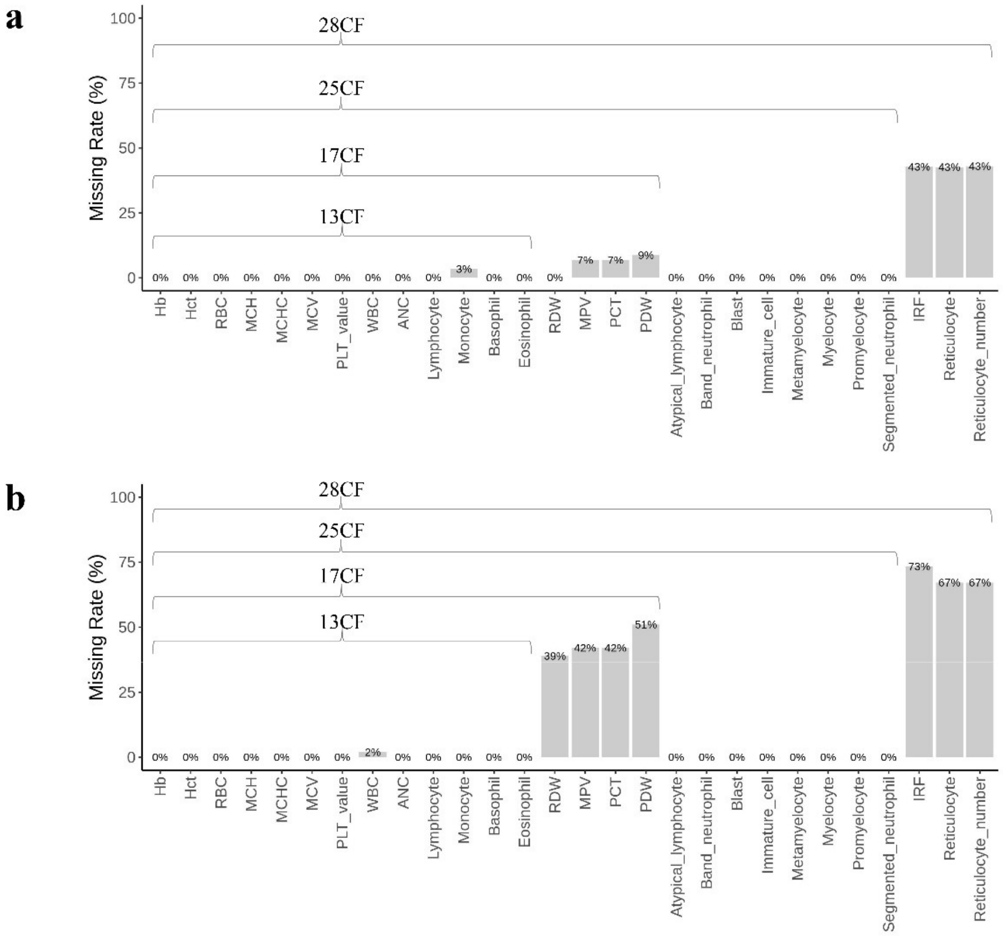

Supplementary Figure 11. Missing Rate of CBC features

Related to Figure 1. Missing rate in (A) SNUH dataset (n=9,044) and (B) CMC dataset (n=17,650). High missing rates are observed for certain features, including IRF, reticulocyte, and reticulocyte number in both the SNUH and CMC datasets, as well as RDW, MPV, PCT, and PDW in the CMC dataset. These high missing rates are a result of the CBC test settings, where these features are not routinely tested.

a

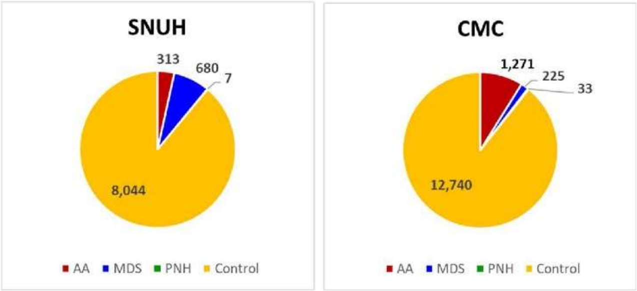

b

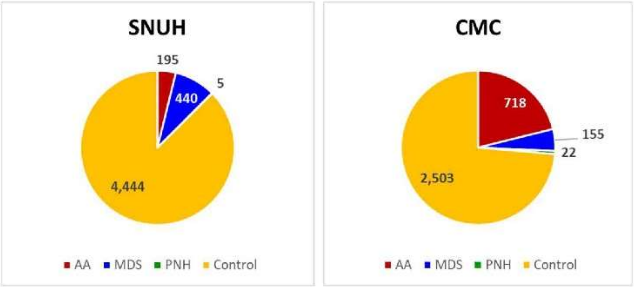

Supplementary Figure 12. Pie Chart of The Number of Patients

Related to Figure 1. (A) The number of whole patients in model development using 13CF, 17CF, 25CF set. (B) The number of patients having CBC records of reticulocytes which are used in 28CF-based classifier development.

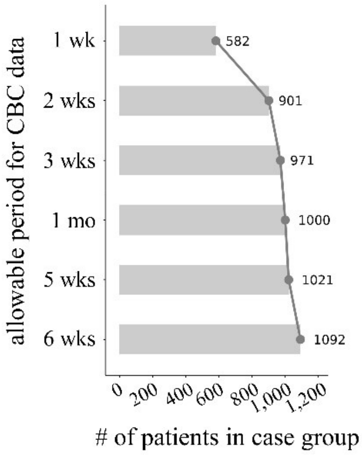

Supplementary Figure 13. Selection of Cutoff Period for CBC Data

Related to Figure 1. The number of patients in the case group for each cutoff period of CBC data based on the diagnostic date. One-month cutoff was selected, allowing for the data from 1000 cases.

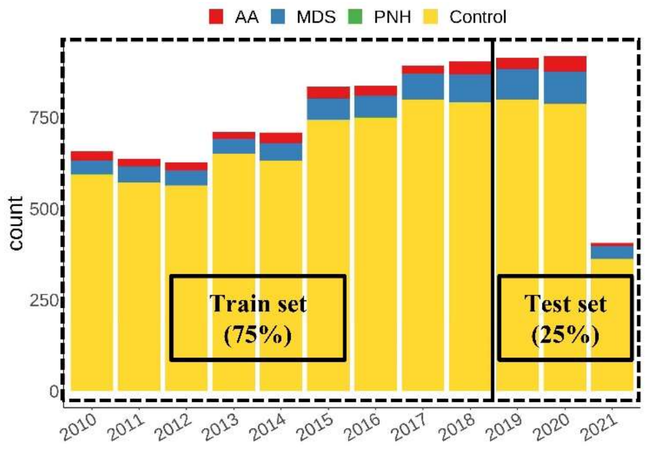

Supplementary Figure 14. Distribution of Diagnosis Dates of SNUH Patients

Related to Figure 1. The training and test sets were split based on the CBC test date. CBCs obtained prior to 2019, which accounted for 75% of the total, were assigned to the training set. The remaining 25% was assigned to the test set.

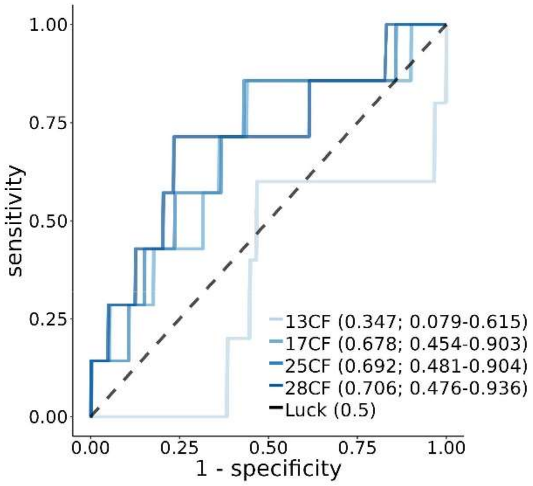

Supplementary Figure 15. Evaluation of an Approach Distinguishing PNH from AA and MDS

Related to Figure 3. ROC curves depicting the performance of binary classifier distinguishing PNH from AA and MDS in the SNUH dataset. The probability of each PNH patient was calculated from the model trained with the remaining PNH patients and AA and MDS patients. The area under the ROC curve (AUROC) is 0.706 or lower, indicating that distinguishing PNH is challenging when using data from insufficient PNH patients.

Supplementary Tables

Feature		CBC Features (CF) 13	17	25	28	BMFS n = 1,559	Control n = 12,740	Fisher's exacttest p-value
Sex (n)	male	o	o	o	o	802	6945	0.0221
	femele					757	5795	

Feature	CBC Features (CF) 13	17	25	28	BMFS n = 1,559 Mean (Median)	Control n = 12,740 Mean (Median)	Wilcoxon test p-value
Age (years)	o	o	o	o	42.43 (44)	48.68 (53)	0
Hb (g/dL)	o	o	o	o	9.35 (9.2)	11.56 (11.5)	3.0976E-214
Hct (%)	o	o	o	o	27.64 (27.1)	34.63 (34.5)	1.2428E-241
RBC (10^6/uL)	o	o	o	o	2.84 (2.81)	3.79 (3.75)	0
RDW (%)		o	o	o	15.92 (15.2)	14.57 (13.9)	1.34534E-26
MCH (pg)	o	o	o	o	33.24 (33.3)	30.66 (30.6)	4.1851E-216
MCHC (g/dL)	o	o	o	o	33.9 (33.9)	33.37 (33.4)	0
MCV (fL)	o	o	o	o	98.14 (98.1)	91.88 (91.6)	5.8321E-149
MPV (fL)		o	o	o	10.27 (10.1)	9.81 (9.7)	3.0086E-14
PCT (%)		o	o	o	0.09 (0.07)	0.25 (0.21)	1.4749E-114
PDW (fL)		o	o	o	11.78 (11.2)	11.04 (10.6)	0.000746018
PLT (10^3/uL)	o	o	o	o	64.1 (41)	224.13 (186.5)	0
WBC (10^3/uL)	o	o	o	o	3.04 (2.79)	11.18 (6.46)	0
ANC (/uL)	o	o	o	o	1322.25 (980)	5942.62 (3580)	0
lymphocyte (% of WBC)	o	o	o	o	51.93 (53)	27.22 (24)	0
monocyte (% of WBC)	o	o	o	o	6.74 (6)	7.94 (7)	0
atypical lymphocyte (%)			o	o	0 (0)	0.03 (0)	0
band neutrophil (%)			o	o	0 (0)	0 (0)	0.620902516
basophil (%)	o	o	o	o	0.29 (0)	0.7 (0)	1.23652E-42
blast (%)			o	o	0.23 (0)	4.47 (0)	0
eosinophil (%)	o	o	o	o	1.18 (0.9)	1.95 (1)	2.48665E-28
immature cell (%)			o	o	0 (0)	0 (0)	0
metamyelocyte (%)			o	o	0.13 (0)	0.63 (0)	0
myelocyte (%)			o	o	0.14 (0)	0.74 (0)	0
promyelocyte (%)			o	o	0 (0)	0.01 (0)	0
segmented neutrophil (% of WBC)			o	o	38.72 (38)	55.57 (59.9)	8.0765E-196

Feature	CBC Features (CF) 13	17	25	28	BMFS n = 895 Mean (Median)	Control n = 2,503 Mean (Median)	Wilcoxon test p-value
IRF (%)				o	15.6 (15.2)	13.33 (11.4)	2.78116E-19
reticulocyte (%)				o	1.64 (1.46)	2.07 (1.65)	0
reticulocyte number (10^9/L)				o	46 (38.9)	75.71 (64.4)	2.18E-69
corrected reticulocyte (%)				o	1.02 (0.86)	1.56 (1.31)	5.74E-51

Supplementary Table 1. CBC Characteristics of the BMFS Patients and Control Group in CMC dataset

Related to Table 1. The feature set column indicates whether the corresponding feature is included in the model. The numbers in the sex row represent the number of patients, and the remaining numbers represent the mean (and median) values of each feature. Corrected reticulocyte is calculated as (patient's Hct/45) * reticulocyte count (%), assuming a normal Hct of 45%. P-values for sex were calculated using Fisher's exact test between the control group and BMFS group. The other p-values were calculated using Wilcoxon test between the control group and BMFS group.

Feature Set	Sex-Stratified Model			Non-Stratified Model (current model)	DeLong's Test
	AUC (male)	AUC (female)	AUC (combined)	AUC	
13CF	0.919 (0.901-0.936)	0.906 (0.886-0.925)	0.912 (0.899-0.925)	0.915 (0.901-0.930)	ns
17CF	0.925 (0.909-0.942)	0.913 (0.894-0.931)	0.919 (0.906-0.931)	0.925 (0.913-0.938)	*
25CF	0.931 (0.915-0.946)	0.918 (0.900-0.936)	0.924 (0.912-0.936)	0.931 (0.919-0.943)	*
28CF	0.931 (0.914-0.947)	0.923 (0.904-0.942)	0.927 (0.914-0.939)	0.936 (0.925-0.948)	*

Supplementary Table 2. Performance Evaluation of Sex-Stratified Models

Related to Figure 3. ROC curves of BMFS classifiers calculated from the sex-stratified model were compared with those of the current models. In the 13CF model, there was no significant difference in AUROC. However, in the 17CF, 25CF, and 28CF models, the current models exhibited slightly higher AUROC than the sex-stratified model. This result suggests that the current models effectively reflect the differences in hematology values related to sex.

A SNUH

feature set		13CF					17CF				
cut-off		100% senssitivity	99% senssitivity	95% senssitivity	90% senssitivity	YouDen index	100% senssitivity	99% senssitivity	95% senssitivity	90% senssitivity	YouDen index
Actual Class	case			294					294		
	control			1950					1950		
Predictive Class	case	1719	1171	901	682	587	1314	1142	846	636	658
	control	525	1073	1343	1562	1657	930	1102	1398	1608	1586
Ture Positive		294	291	280	265	257	294	291	280	265	270
False Positive		1425	880	621	417	330	1020	851	566	371	388
Ture Negative		525	1070	1329	1533	1620	930	1099	1384	1579	1562
False Negatie		0	3	14	29	37	0	3	14	29	24
sensitivity		100.0% (294/294)	99.0% (291/294)	95.2% (280/294)	90.1% (265/294)	87.4% (257/294)	100.0% (294/294)	99.0% (291/294)	95.2% (280/294)	90.1% (265/294)	91.2% (268/294)
specificity		26.9% (525/1950)	54.9% (1070/1950)	68.2% (1329/1950)	78.6% (1533/1950)	83.1% (1620/1950)	47.7% (930/1950)	55.4% (1080/1950)	70.8% (1381/1950)	81.0% (1579/1950)	80.4% (1568/1950)
PPV		17.1% (294/1719)	24.9% (291/1171)	31.1% (280/901)	38.9% (265/682)	43.8% (257/587)	22.4% (294/1314)	25.1% (291/1161)	33.0% (280/849)	41.7% (265/636)	41.2% (268/650)
NPV		100.0% (294/525)	99.7% (1070/1073)	99.0% (1329/1343)	98.1% (265/1562)	97.8% (1620/1657)	100.0% (294/930)	99.7% (1080/1083)	99.0% (1381/1395)	98.2% (265/1608)	98.4% (1568/1594)

feature set		25CF					28CF				
cut-off		100% senssitivity	99% senssitivity	95% senssitivity	90% senssitivity	YouDen index	100% senssitivity	99% senssitivity	95% senssitivity	90% senssitivity	YouDen index
Actual Class	case			294					278		
	control			1950					1781		
Predictive Class	case	1224	1044	810	617	663	1056	976	641	570	474
	control	1020	1200	1434	1627	1581	1003	1083	1418	1489	1585
Ture Positive		294	291	280	265	273	278	276	264	251	242
False Positive		930	753	530	352	390	778	700	377	319	232
Ture Negative		1020	1197	1420	1598	1560	1003	1081	1404	1462	1549
False Negatie		0	3	14	29	21	0	2	14	27	36
sensitivity		100.0% (294/294)	99.0% (291/294)	95.2% (280/294)	90.1% (265/294)	92.9% (273/294)	100.0% (278/278)	99.3% (276/278)	95.0% (264/278)	90.3% (251/278)	87.1% (242/278)
specificity		52.3% (1020/1950)	61.4% (1197/1950)	72.8% (1420/1950)	81.9% (1598/1950)	80.0% (1560/1950)	56.3% (1003/1781)	60.7% (1081/1781)	78.8% (1404/1781)	82.1% (1462/1781)	87.0% (1549/1781)
PPV		24.0% (294/1224)	27.9% (291/1044)	34.6% (280/810)	42.9% (265/617)	41.2% (273/663)	26.3% (278/1056)	28.3% (276/976)	41.2% (264/641)	44.0% (251/570)	51.1% (242/474)
NPV		100.0% (294/1020)	99.8% (1197/1200)	99.0% (1420/1434)	98.2% (265/1627)	98.7% (1560/1581)	100.0% (278/1003)	99.8% (276/1083)	99.0% (264/1418)	98.2% (251/1489)	97.7% (242/1585)

B CMC

feature set	13CF					17CF				
cut-off	100% senssitivity	99% senssitivity	95% senssitivity	90% senssitivity	YouDen index	100% senssitivity	99% senssitivity	95% senssitivity	90% senssitivity	YouDen index
Actual Class case			1559					1559		
Actual Class control			12740					12740		
Predictive Class case	12031	8317	5998	3836	3184	9560	8188	5488	3640	3763
Predictive Class control	2268	5982	8301	10463	11115	4739	6111	8811	10659	10536
Ture Positive	1553	1532	1492	1409	1350	1543	1532	1479	1408	1417
False Positive	10478	6785	4506	2427	1834	8017	6656	4009	2232	2346
Ture Negative	2262	5955	8234	10313	10906	4723	6084	8731	10508	10394
False Negatie	6	27	67	150	209	16	27	80	151	142
sensitivity	99.6% (1553/1559)	98.3% (1532/1559)	95.7% (1492/1559)	90.4% (1409/1559)	86.6% (1350/1559)	99.0% (1543/1559)	98.3% (1532/1559)	94.9% (1479/1559)	90.3% (1408/1559)	90.9% (1417/1559)
specificity	17.8% (2262/12740)	46.7% (5955/12740)	64.6% (8234/12740)	80.9% (10313/12740)	85.6% (10906/12740)	37.1% (4723/12740)	47.8% (6084/12740)	68.5% (8731/12740)	82.5% (10508/12740)	81.6% (10394/12740)
PPV	12.9% (1553/12031)	18.4% (1532/8317)	24.9% (1492/5998)	36.7% (1409/3836)	42.4% (1350/3184)	16.1% (1543/9560)	18.7% (1532/8188)	26.9% (1479/5488)	38.7% (1408/3640)	37.7% (1417/3763)
NPV	99.7% (1553/2268)	99.5% (1532/5982)	99.2% (1492/8301)	98.6% (1409/10463)	98.1% (1350/11115)	99.7% (1543/4739)	99.6% (1532/6111)	99.1% (1479/8811)	98.6% (1408/10659)	98.7% (1417/10536)

feature set	25CF					28CF				
cut-off	100% senssitivity	99% senssitivity	95% senssitivity	90% senssitivity	YouDen index	100% senssitivity	99% senssitivity	95% senssitivity	90% senssitivity	YouDen index
Actual Class case			1559					895		
Actual Class control			12740					2503		
Predictive Class case	8687	7311	5022	3320	3679	2344	2167	1299	1198	1072
Predictive Class control	5612	6988	9277	10979	10620	1054	1231	2099	2200	2326
Ture Positive	1539	1518	1469	1398	1419	888	883	836	816	776
False Positive	7148	5793	3553	1922	2260	1456	1284	463	382	296
Ture Negative	5592	6947	9187	10818	10480	1047	1219	2040	2121	2207
False Negatie	20	41	90	161	140	7	12	59	79	119
sensitivity	98.7% (1539/1559)	97.4% (1518/1559)	94.2% (1469/1559)	89.7% (1398/1559)	91.0% (1419/1559)	99.2% (888/895)	98.7% (883/895)	93.4% (836/895)	91.2% (816/895)	86.7% (776/895)
specificity	43.9% (5592/12740)	54.5% (6947/12740)	72.1% (9187/12740)	84.9% (10818/12740)	82.3% (10480/12740)	41.8% (1047/2503)	48.7% (1219/2503)	81.5% (2040/2503)	84.7% (2121/2503)	88.2% (2207/2503)
PPV	17.7% (1539/8687)	20.8% (1518/7311)	29.3% (1469/5022)	42.1% (1398/3320)	38.6% (1419/3679)	37.9% (888/2344)	40.7% (883/2167)	64.4% (836/1299)	68.1% (816/1198)	72.4% (776/1072)
NPV	99.6% (1539/5612)	99.4% (1518/6988)	99.0% (1469/9277)	98.5% (1398/10979)	98.7% (1419/10620)	99.3% (888/1054)	99.0% (883/1231)	97.2% (836/2099)	96.4% (816/2200)	94.9% (776/2326)

Supplementary Table 3. Confusion matrix and Evaluation metrics

Related to Figure 3. The confusion matrix and evaluation metrics for the classifier performance in the (A) SNUH dataset and (B) CMC dataset. The table includes true positive (TP), false positive (FP), true negative (TN), and false negative (FN) values, as well as corresponding values for sensitivity, specificity, positive predictive value (PPV), and negative predictive value (NPV).

A SNUH

	cut-offs				
	100% sensitivity	99% sensitivity	95% sensitivity	90% sensitivity	YouDen index
13CF	100% (7/7)	100% (7/7)	100% (7/7)	85.7% (6/7)	71.4% (5/7)
17CF	100% (7/7)	100% (7/7)	100% (7/7)	85.7% (6/7)	85.7% (6/7)
25CF	100% (7/7)	100% (7/7)	100% (7/7)	85.7% (6/7)	85.7% (6/7)
28CF	100% (5/5)	100% (5/5)	100% (5/5)	80.0% (4/5)	80.0% (4/5)

B CMC

	cut-offs from SNUH				
	100% sensitivity	99% sensitivity	95% sensitivity	90% sensitivity	YouDen index
13CF	97.0% (32/33)	97.0% (32/33)	93.9% (31/33)	78.8% (26/33)	72.7% (24/33)
17CF	97.0% (32/33)	97.0% (32/33)	93.9% (31/33)	81.8% (27/33)	87.9% (29/33)
25CF	97.0% (32/33)	97.0% (32/33)	93.9% (31/33)	81.8% (27/33)	84.8% (28/33)
28CF	100% (22/22)	95.5% (21/22)	86.7% (19/22)	77.3% (17/22)	72.7% (16/22)

Supplementary Table 4. Prediction Results of PNH patients

Related to Figure 3. (A) Sensitivity of predicting PNH as BMFS in the (A) SNUH and (B) CMC dataset. The cut-off probabilities are set based on the results of the BMFS classifier in the SNUH dataset.

SNUH	CMC
Sysmex XE-2100	Sysmex XE-2100
Sysmex XN-9000 XN10	Sysmex XN-9000 XN10
Sysmex XN-9000 XN20	Sysmex XN-9000 XN20
Sysmex XN-9100 XN10	Sysmex XN-1500 XN20
Sysmex XN-9100 XN20	Beckman coulter DXH800
SIEMENS ADVIA 2120i	

Supplementary Table 5. List of CBC Analysers

Related to Figure 1.

class	ICD-10 code	ICD-10 name	%
AA	D610	Constitutional aplastic anaemia	0.1%
	D611	Drug-induced aplastic anaemia	<0.1%
	D612	Aplastic anaemia due to other external agents	<0.1%
	D613	Idiopathic aplastic anaemia	0.6%
	D618	Other specified aplastic anaemias	0.1%
	D619	Aplastic anaemia, unspecified	2.7%
MDS	D461	Refractory anaemia with ring sideroblasts	0.1%
	D462	Refractory anaemia with excess of blasts	0.1%
	D466	Myelodysplastic syndrome with isolated del(5q) chromosomal abnormality	0.1%
	D467	Other myelodysplastic syndromes	<0.1%
	D469	Myelodysplastic syndrome, unspecified	7.3%
PNH	D595	Paroxysmal nocturnal haemoglobinuria [Marchiafava-Micheli]	0.1%
Control	C819	Hodgkin lymphoma, unspecified	1.4%
	C829	Follicular lymphoma, unspecified	1.7%
	C833	Diffuse large B-cell lymphoma	9.9%
	C844	Peripheral T-cell lymphoma, not classified	1.5%
	C859	Non-Hodgkin lymphoma, unspecified	10.7%
	C884	Extranodal marginal zone B-cell lymphoma of mucosa-associated lymphoid tissue [MALT-lymphoma]	1.9%
	C900	Multiple myeloma	8.1%
	C910	Acute lymphoblastic leukaemia [ALL]	1.4%
	C920	Acute myeloblastic leukaemia [AML]	3.8%
	C921	Chronic myeloid leukaemia[CML], BCR/ABL-positive	1.9%
	C950	Acute leukaemia of unspecified cell type	2.0%
	D472	Monoclonal gammopathy of undetermined significance (MGUS)	3.4%
	D473	Essential(haemorrhagic) thrombocythaemia	2.3%
	D649	Anaemia, unspecified	3.0%
	D696	Thrombocytopenia, unspecified	2.4%
	D728	Other specified disorders of white blood cells	1.6%
	D758	Other specified diseases of blood and blood-forming organs	2.3%
	R899	Unspecified abnormal finding in specimens from other organs, systems and tissues	1.9%
	the others		27.5%

Supplementary Table 6. ICD-10 Code of Case Group

Related to Figure 1.
